# Editorial: Clinical applications of artificial intelligence in retinal and optic nerve disease

**DOI:** 10.3389/fmed.2022.1065603

**Published:** 2022-11-01

**Authors:** Zhichao Wu, Linda M. Zangwill, Felipe A. Medeiros, Tiarnan D. L. Keenan

**Affiliations:** ^1^Centre for Eye Research Australia, Royal Victorian Eye and Ear Hospital, East Melbourne, VIC, Australia; ^2^Ophthalmology, Department of Surgery, University of Melbourne, Melbourne, VIC, Australia; ^3^Viterbi Family Department of Ophthalmology, Hamilton Glaucoma Center, University of California, San Diego, La Jolla, CA, United States; ^4^Department of Ophthalmology, Duke Eye Center, Duke University School of Medicine, Durham, NC, United States; ^5^Division of Epidemiology and Clinical Applications, National Eye Institute, National Institutes of Health, Bethesda, MD, United States

**Keywords:** artificial intelligence (AI), retina, optic nerve, age-related macular degeneration, glaucoma, imaging, diabetic retinopathy, natural language processing

Recent estimates indicate that retinal and optic nerve conditions such as age-related macular degeneration (AMD), glaucoma and diabetic retinopathy (DR) are the causes of irreversible blindness or moderate and severe visual impairment in over 20 million people worldwide (1). Our ability to prevent irreversible vision loss from these conditions (and others, such as inherited retinal diseases) are significantly hindered by challenges faced with their clinical management, and/or in the discovery of new therapies. These include challenges in the accurate and early detection of the disease and the prediction of disease progression or visual prognosis following treatment. These also include challenges in having sufficiently robust outcome measures for trials of new interventions, as well as the pragmatic issue of expediently identifying eligible individuals to offer participation in such trials.

Recent advances in artificial intelligence (AI), especially a subset of machine learning techniques termed *deep learning* (2), show promise for addressing these challenges to enable the prevention of irreversible vision loss (3–6). For instance, Zhang et al. conducted a prospective trial to evaluate the feasibility and performance of a deep learning-driven software that provides an automatic grading of DR severity based on color fundus photographs and demonstrated that it had an area under the receiving operating characteristic curve (AUC) of 0.96 for detecting referrable DR, based on the ground truth of manual grading by ophthalmologists. Huang et al. developed a multimodal deep learning model to detect eyes that were clinically diagnosed as having glaucoma using fundus photographs and visual field results obtained from a device that performs fundus-oriented perimetry with a scanning laser ophthalmoscope. They reported that this model using combined imaging and visual function inputs had an AUC of 0.97, outperforming models using either modality alone.

Wang and Birch utilized deep learning to segment the ellipsoid zone (EZ) on optical coherence tomography (OCT) scans of eyes with retinitis pigmentosa (RP), a band that is closely associated with visual sensitivity losses. Their new deep learning segmentation model had an average Dice similarity coefficient of 0.87, compared to manual annotations of the EZ area, showing promise for significantly reducing the cost and burden of manual measurements by reading centers when evaluating novel therapies for this condition. Bogunović et al. also used deep learning approaches to segment imaging biomarkers from OCT scans at the baseline and 4-week follow-up visit of eyes undergoing treatment for neovascular AMD to predict long-term visual outcomes. These outcomes were determined based on an analysis of longitudinal visual acuity measurements using a latent class mixed model to classify eyes as being “responders” or “non-responders”. A prediction model utilizing the quantified OCT imaging biomarkers, along with clinical and demographic data, was then developed for this visual function outcome using a machine learning approach (a random forest classifier), and this model had an AUC of 0.87 for predicting the outcome based on visual acuity trajectories.

Each of these studies demonstrates the potential utility of AI in the automated detection of retinal and optic nerve diseases, and quantification of key disease features on imaging needed to improve the feasibility of new treatment trials and for personalized management. Follow-up studies are now needed to further examine the clinical significance of these newly developed AI approaches by evaluating their performance at directly predicting clinical outcomes that reflect “*how a patient feels, functions, or survives*” (7) (as opposed to merely predicting disease or anatomical features on retinal imaging). Finally, Chen and Baxter reviewed how AI could be used—beyond its common application in image-based deep learning—for natural language processing. This approach may be especially useful for identifying potentially eligible individuals from electronic health records that may meet the criteria for clinical trials—especially for rarer inherited eye diseases—thus facilitating their conduct and expediting treatment discovery.

Together, advances in AI could continue to help us make significant progress in reducing the global burden of irreversible vision loss, and we hope the articles in this Research Topic facilitate this progress within our field and beyond.

## Author contributions

All authors listed have made a substantial, direct, and intellectual contribution to the work and approved it for publication.

## Conflict of interest

LZ: financial support–National Eye Institute, Carl Zeiss Meditec, Inc, Heidelberg Engineering, Optovue, Inc, Topcon Medical System, Inc. Consultant, IDx, and Allergan. FM: Aeri Pharmaceuticals, Allergan, Annexon, Biogen, Carl Zeiss Meditec, Galimedix, Stealth Biotherapeutics, Stuart Therapeutics, and Reichert, financial support–Allergan, Carl Zeiss Meditec, Google, Inc., Heidelberg Engineering, Novartis, and Reichert, and Patent–nGoggle, Inc. TK: patent application for Methods and systems for predicting rates of progression of age-related macular degeneration. The remaining author declares that the research was conducted in the absence of any commercial or financial relationships that could be construed as a potential conflict of interest.

## Publisher's note

All claims expressed in this article are solely those of the authors and do not necessarily represent those of their affiliated organizations, or those of the publisher, the editors and the reviewers. Any product that may be evaluated in this article, or claim that may be made by its manufacturer, is not guaranteed or endorsed by the publisher.
